# Health-promoting housing policy in a changing climate: integrating affordability, security, and resilience

**DOI:** 10.1093/heapro/daaf238

**Published:** 2026-01-13

**Authors:** Martin McKee, Philippa Howden-Chapman, Isobel Braithwaite, Rebecca Bentley

**Affiliations:** Department of Health Services Research and Policy, London School of Hygiene & Tropical Medicine, London, United Kingdom; He Kāinga Oranga/Housing and Health Research Programme, University of Otago, PO Box 7343, Newtown, Wellington 6242, New Zealand; Public Health Division, London Borough of Tower Hamlets, Tower Hamlets Town Hall, 160 Whitechapel Road, London E1 1BJ, United Kingdom; NHMRC Centre of Research Excellence in Healthy Housing, 207-221 Bouverie St, Melbourne, Victoria 3010, Australia

**Keywords:** homelessness, healthy communities, healthy public policy, healthy settings, inequalities

## Abstract

Housing is a fundamental determinant of health, yet many housing systems fail to promote well-being or address growing challenges such as climate change, inequality, and urbanization. It is increasingly treated as an investment vehicle and as a commercial product supporting the construction industry, with substantial interactions with climate change, rather than its fundamental role providing shelter. This perspective addresses this gap by proposing an integrated framework for health-promoting housing policy that combines affordability, security, and quality, paying particular attention to their interdependence and the growing influence of climate, adopting a systems-thinking approach. The framework was developed through an iterative literature synthesis and interdisciplinary dialogue, designed to overcome the disciplinary fragmentation of existing evidence. We conducted structured searches in PubMed, Scopus, and Web of Science, supplemented by grey literature, concentrating on materials published since 2010, reflecting the increasing relevance of climate resilience and equity. Inclusion criteria focused on sources examining housing–health interactions, policy interventions, and systemic challenges; purely technical engineering studies were excluded. Themes were mapped against four policy levers, legal, financial, planning, and community-based measures. The resulting framework offers policymakers a flexible menu of options to strengthen housing systems and advance health equity. By integrating climate resilience and social inclusion into housing policy, this approach provides a foundation for coordinated action across sectors. Aligning housing policy with public health goals is essential for building equitable, sustainable, and resilient communities.

Contribution to Health Promotion

- Good housing protects against illness by reducing damp, mould, cold, and overcrowding.
- It supports mental well-being through secure, stable, and affordable living conditions.
- Healthy homes help people stay warm in winter and cool in summer.
- Well-designed neighbourhoods encourage physical activity and social connection.
- Climate-resilient housing reduces health risks from floods, heatwaves, and storms.

## Introduction

Good health requires good housing. Bentley *et al.* (2025) have described how a healthy housing policy is built on three pillars: affordability, security, and suitability. These, in turn, are influenced by diverse socio-economic conditions, including income, employment, and education. Yet many housing policies undermine rather than promote health (Howden-Chapman *et al.* 2023). Getting housing policy right is more important than ever in a world increasingly affected by climate change, where population movements are occurring faster than housing can keep pace, leaving too many people in substandard homes and informal settlements.

The pathways linking housing and health are many and complex. Poor quality accommodation, with inadequate ventilation, damp, and poor insulation, contributes to temperature-related illness and respiratory and cardiovascular diseases (Sims *et al.* 2020, Wimalasena *et al.* 2021). Overcrowding facilitates infectious disease transmission (Baker *et al.* 2000). Substandard housing increases the risks of injury (Gallagher *et al.* 2024) and child maltreatment (Chandler *et al.* 2022). Insecure housing increases mental health disorders (Gili *et al.* 2013, Clair *et al.* 2016a, 2016b, Mason *et al.* 2024), while unaffordable housing exacerbates inequality and homelessness (Bentley *et al.* 2025).

All these pathways and others have been studied in depth, along with legal, financial, and regulatory approaches to the creation of healthier housing. Yet much of this work remains in disciplinary siloes despite its interconnectedness. Academic structures, including funding streams and disciplinary boundaries, discourage the holistic approach needed to synthesize all relevant insights.

This paper rejects that reductionist model. It is written for policymakers in housing and health, based on the premise that they need not be experts but should grasp key concepts and evidence on what works. It is structured around Bentley *et al*.’s pillars, affordability, security, and suitability, followed by sections on climate change, informal settlements, and hopelessness. Adopting a global perspective, it recognizes that while some concepts are universal, many require contextual tailoring. Accordingly, it avoids prescriptive recommendations and instead offers adaptable policy options.

## Methods

We undertook an iterative literature review and interdisciplinary dialogue to develop a conceptual model that synthesizes evidence on housing policy and its intersections with public health, climate resilience, and equity. We combined structured searches with adaptive exploration, using major databases such as PubMed, Scopus, and Web of Science with evolving search terms. Searches were limited to publications from 2010 onwards to reflect the growing relevance of climate resilience and equity, though a few seminal earlier references were included. We also conducted focused searches on Google Scholar as necessary. Sources included peer-reviewed articles, policy documents, and grey literature addressing housing as a determinant of health or its links with climate, equity, or governance, including WHO’s Health and Housing guidelines (World Health Organization 2018) and key policy exemplars. Studies focused solely on technical engineering without health or policy relevance were excluded. No human subjects were involved so ethics approval was not required.

The research team comprised experts in public health, housing policy, urban planning, and health economics, with global experience. Interdisciplinary dialogue was central to the process as our framework integrates findings from epidemiology, economics, planning, legal scholarship, systems analysis, and implementation case studies (Nilsson *et al.* 2018). Structured discussions amongst the authors, drawing on their varied expertise, helped interpret findings, identify gaps, and refine policy options, including a practical checklist for health promotion in housing. This iterative process ensured the framework was both evidence informed and adaptable to different governance and socio-political contexts. While not exhaustive, it offers policymakers a strategic menu of options suited to diverse global housing challenges.

## Results

**Figure 1:**
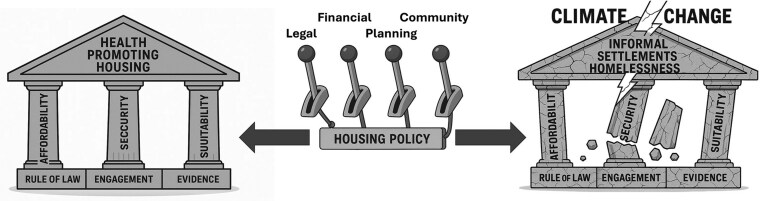
Conceptual framework for health-promoting housing. Source: authors’ compilation.

As noted, our framework builds on and extends Bentley *et al.*’s (2025) work and that of Li *et al.* (2025). Each of their three pillars can be strengthened through policy levers: regulatory and legal tools, fiscal measures, planning and infrastructure, and participative community initiatives. Three foundational elements underpin the framework: rule of law and good governance, evidence-based policy, and population empowerment and engagement. When these fail, informal settlements and homelessness emerge, and climate change increasingly threatens structural integrity. Together, these components provide a comprehensive framework for evaluating and shaping housing policy to promote health, equity, and sustainability. Figure 1 presents the framework visually. We now discuss each pillar and the policy measures to reinforce them, followed by sections on climate change, informal settlements, and homelessness.

### Affordability

For many people, housing costs are their single largest expenditure and determine what they can afford for everything else needed to live healthy and fulfilling lives. At its simplest, housing affordability is determined by a combination of market forces and policy choices and is thus, to a certain extent, a function of demand and supply. Greater demand, e.g. as a consequence of population growth and changes in household composition, requires a greater supply, in the form of increased property development (Andrews 2010, Gleeson 2023). Yet, supply frequently fails to keep pace with demand, so prices rise (Phillips 2020). In this section, we aim to explain why this is the case and what can be done to address it.

#### Legal and regulatory tools

Legal and regulatory tools play a key role in addressing structural imbalances that affect housing affordability. A major issue is information asymmetry, when developers, buyers, and sellers have unequal knowledge about property conditions or neighbourhoods, leading to poor decisions. To address this, many jurisdictions require disclosure of key details, such as structural reports and energy ratings, prior to sale. Evidence from high-income countries (Nanda and Ross 2012, Frondel *et al.* 2020) and India (Tandel *et al.* 2025) suggests that such measures can improve transparency and efficiency, though their impact depends on clarity and accessibility (Waye *et al.* 2023) and may be less effective for characteristics that buyers can easily observe (Cassidy 2022).

Standardizing valuation and inspection protocols can improve consistency in property assessments. Given the uniqueness of each property (Brennan *et al.* 2017), oversight of estate agents and surveyors, along with strong consumer protection laws, can reduce misrepresentation and build trust.

Power asymmetry is another challenge, especially where large developers or landlords dominate. They may hoard land to inflate prices (Guthrie 2023). Competition law and antilobbying regulations (Anderson *et al.* 2025, Jacobs 2015) can help curb monopolistic practices and undue influence (Mata *et al.* 2015, Canada Green Building Council 2021).

Legal measures can also require developers to contribute to public infrastructure, internalizing social and environmental costs (Gielen and van der Krabben 2019). Additionally, transaction costs, such as legal fees and taxes, can deter mobility, especially for lower-income households, potentially distorting labour markets. While evidence from England questions this link (Hilber and Lyytikäinen 2017), reducing such costs remains a relevant strategy for improving affordability.

#### Fiscal and financial measures

Fiscal and financial policy tools are critical in determining housing affordability, particularly in contexts where market mechanisms alone fail to deliver equitable outcomes. These measures can influence both the demand and supply sides of the housing market, helping to curb speculative behaviour, incentivize inclusive development, and support vulnerable populations.

One of the most pressing challenges is the financialization of housing, where property is increasingly treated as an investment vehicle rather than a place to live (García-Lamarca and Kaika 2016). This shift has encouraged speculative practices that inflate prices and reduce access to shelter for those seeking it. To counter this, governments can implement capital gains taxes and/or other taxes on second homes, with differential treatment of primary residences to avoid penalizing owner-occupiers. Similarly, land value taxes can discourage the hoarding of buildable land, ensuring that approved development sites are used efficiently and not withheld to manipulate market prices (Guthrie 2023).

Governments also play a direct role in funding housing supply, particularly through the construction of public or social housing. Cities such as Vienna, Helsinki, and Singapore have demonstrated the success of such interventions, showing that well-designed public housing can be both affordable and high quality (Cram 2015, OECD 2021, Kadi and Lilius 2024). In addition to new builds, fiscal measures can support the redevelopment and upgrading of substandard housing, particularly in areas with surplus stock that is underutilized due to poor conditions, crime, or inadequate infrastructure.

Subsidies and tax incentives are another key tool. These can be used to encourage inclusive redevelopment schemes, promote energy-efficient upgrades, or support low-income households in accessing adequate housing. Such measures are particularly important in areas undergoing regeneration, where rising property values risk displacing long-standing residents.

Together, these fiscal and financial strategies offer governments a powerful means of shaping housing markets to better serve public health and social equity goals. When deployed thoughtfully, they can help rebalance the housing system, making it more resilient, inclusive, and responsive to the needs of all communities.

#### Planning and infrastructure measures

Planning and infrastructure policies are fundamental to ensuring that housing supply meets demand in ways that promote affordability, sustainability, and public health. Greater demand, e.g. as a consequence of population growth and changes in household composition, requires a greater supply, in the form of increased property development (Andrews 2010, Gleeson 2023). Yet, housing supply remains notoriously inelastic. Long construction timelines, restrictive planning regulations, and land scarcity all contribute to sluggish responsiveness to changing demand. These constraints often lead to price volatility and persistent shortages, particularly when changes in the nature of the workforce stimulate internal migration.

Housing decisions frequently generate externalities, unintended social and environmental consequences that are rarely accounted for in market transactions (Rossi-Hansberg and Sarte 2012). As noted above, new developments can trigger gentrification, displacing lower-income residents as property values rise (Chapple 2017). Poorly planned projects, including both low- and high-density developments, can worsen traffic, increase greenhouse gas emissions, reduce air quality, or negatively impact the quality of public spaces (Ewing *et al.* 2002).

To mitigate these effects, governments can require developers to offset the social and environmental costs of their activities. This might include obligations to invest in civic amenities, affordable housing, or public infrastructure such as transport links and green spaces, assets that benefit both new and existing residents (Gielen and van der Krabben 2019). Such mechanisms help internalize the broader impacts of development and ensure that growth contributes positively to community well-being.

Strategic planning frameworks can guide urban growth to balance density with liveability, prioritizing walkability, access to services, and environmental resilience. These frameworks require strong governance and cross-sector coordination across housing, transport, and environmental planning.

Housing policy must move beyond quantity to embrace quality, equity, and integration. New developments should integrate with transport, health, education, and green space systems to enable communities to thrive. Ultimately, planning and infrastructure are not just about building homes but creating better communities. When aligned with public health and equity goals, these tools can deliver housing systems that are inclusive, resilient, and responsive.

#### Community initiatives and participatory approaches

Community-led strategies and participatory planning complement legal and regulatory measures for housing affordability. They recognize housing as a cornerstone of social well-being and empower communities to correct market imbalances and foster inclusive development. Financial literacy programmes help buyers and renters navigate housing markets by simplifying contracts, financing options, and property rights, reducing vulnerability to exploitation. Governments can also support small- and medium-sized enterprises (SMEs) in development and supply chains by improving access to credit and offering training. This diversifies housing supply, reduces reliance on large firms, and promotes locally responsive practices (Tapp and Peiser 2023).

Equally vital is the role of participatory planning. Inclusive consultation mechanisms, particularly those that engage marginalized communities, can help ensure that housing developments reflect local needs and values. Such approaches are especially important in mitigating the negative externalities of urban development, including the consequences of gentrification, which can lead to exclusion and displacement (Chapple 2017). When communities are meaningfully involved in shaping their environments, the resulting policies and projects are more likely to be equitable, sustainable, and resilient.

### Security

Housing security refers to the certainty of tenure in a home, i.e. ‘not having to worry about being evicted or having your home or lands taken away’ (United Nations Human Rights 2025). Conversely, housing insecurity encompasses a range of issues from precarious rental agreements to the risk of eviction (Chisholm *et al.* 2022). Housing insecurity significantly impacts mental and physical health (Clair *et al.* 2016a, 2016b) and wider social well-being. In the context of evictions from rental housing, e.g. it has been found to deepen poverty, impede healthcare access, lower adherence to Human Immunodeficiency Virus (HIV) medication, and impose food insecurity (Smith *et al.* 2024). There is also evidence that it is associated with adverse birth outcomes and impaired cognitive development in children whose parents are evicted, and recurrence or initiation of substance abuse amongst adults, via a range of negative impacts on mental health (Ramphal *et al.* 2023). While housing insecurity can be driven by unaffordability, as discussed in the previous section, we focus the following discussion on policies that address other aspects of housing security. The consequences of failure are discussed in a separate section, later in this paper, which addresses the impacts of homelessness.

Housing insecurity can arise for several reasons. One is insecurity of tenure due to inadequate protection against eviction for tenants and gaps in social safety nets, such as housing benefits and mortgage support (Reeves *et al.* 2016) for those experiencing job loss or other shocks (Ramphal *et al.* 2023, Smith *et al.* 2024). Another is insecurity resulting from social instability or crises, e.g. in locations experiencing conflict, criminal activity, or civil unrest, or the consequences of intimate partner violence (Klein *et al.* 2021).

A third is the risk of exposure to harmful temperatures, disasters, and extreme weather events, whose frequency and severity are exacerbated by climate change (Bezgrebelna *et al.* 2021). These last risks can manifest as displacement due to climatic events and as an inability to obtain property insurance. While these climate-related threats are explored in greater detail later in this paper, it is important to note here that they directly intersect with housing security, particularly for populations living in vulnerable or poorly protected areas.

#### Legal and regulatory tools

Legal protections are central to reducing housing insecurity, particularly for renters experiencing unstable tenure or facing eviction. Strengthening these safeguards improves housing stability and health. Key interventions include reforming tenancy laws to require longer leases, cause for eviction, and extended notice periods, helping tenants plan with certainty (Whitehead *et al.* 2019). Rent controls remain debated but can work within broader packages that include investment in affordable housing and enforcement of standards, balancing tenant and landlord interests (Whitehead *et al.* 2019). During crises such as COVID-19, eviction moratoria and mortgage protections have been vital in preventing displacement and its adverse health impacts (Chen *et al.* 2022).

More broadly, social safety nets, including housing benefits and mortgage support, are essential for individuals and families facing economic shocks (Reeves *et al.* 2016). These supports can buffer against the immediate impacts of job loss or illness, helping to maintain housing stability during periods of financial hardship (Ramphal *et al.* 2023, Smith *et al.* 2024).

The importance of these legal and regulatory tools is underscored by the growing body of evidence linking housing insecurity to adverse health outcomes. These include increased rates of mental distress, food insecurity, and poor physical health, as well as developmental impacts on children and heightened vulnerability to substance abuse (Clair *et al.* 2016a, 2016b, Ramphal *et al.* 2023, Smith *et al.* 2024). By reinforcing legal protections and ensuring access to support, governments can play a decisive role in mitigating these risks and promoting housing as a foundation for health.

#### Fiscal and financial measures

Financial interventions are vital for addressing housing insecurity, particularly amongst individuals experiencing economic hardship or sudden shocks. While evidence on some measures is mixed, fiscal tools can still offer crucial support and stability for vulnerable households.

Emergency rent assistance is one of the most direct approaches, helping tenants avoid eviction during financial crises. Other supports, such as legal aid, priority access to public housing, and long-term rent subsidies, can also enhance housing security, though their effectiveness varies by context and implementation (Chen *et al.* 2022).

For homeowners, mortgage support schemes are important during times of crisis. These programmes can prevent foreclosure and displacement, particularly when households experience job loss, illness, or other destabilizing events (Ramphal *et al.* 2023, Smith *et al.* 2024). By cushioning the impact of economic shocks, such supports help maintain housing stability and reduce the risk of cascading health and social consequences.

More broadly, social safety nets, including housing benefits, play a foundational role in protecting individuals and families from the worst effects of housing insecurity. These financial supports are especially important in systems where market forces alone fail to ensure equitable access to safe and stable housing.

Although not all fiscal measures have a robust evidence base, their potential to mitigate harm and promote resilience should at least warrant consideration in any comprehensive housing policy strategy.

#### Planning and infrastructure measures

Urban planning and infrastructure are key to housing security, shaping environments for long-term stability and resilience. While legal and financial tools address immediate risks, planning creates inclusive communities that support well-being. A core goal is designing walkable neighbourhoods that foster social interaction and informal support networks. Such environments promote physical activity and mental health and help buffer residents against isolation and vulnerability linked to housing insecurity (Ramos-Vidal and de la Ossa 2024).

Planning must also respond to broader social and environmental threats. These include social instability or crises, e.g. in locations experiencing conflict, criminal activity, or civil unrest, or the consequences of intimate partner violence (Klein *et al.* 2021). Similarly, climate-related risks, such as extreme weather events, rising temperatures, and flooding, pose growing threats to housing security. These risks can lead to displacement or make it difficult for residents to obtain property insurance, further undermining tenure stability (Bezgrebelna *et al.* 2021). This is only one example of the impact of climate change on housing and health; the topic is explored further later in this paper.

To address these challenges, planning must integrate housing policy with climate-resilient infrastructure. This includes zoning to restrict development in high-risk areas, investment in protective systems, and cross-sector coordination to ensure that housing is linked to transport, health, and community services.

Ultimately, planning should treat housing as a public good that supports health, equity, and sustainability. When combined with participatory governance and robust legal frameworks, these tools can foster communities in which housing security is a shared foundation for well-being.

#### Community initiatives and participatory approaches

Community-led initiatives and participatory governance are vital components of a housing system that promotes security and equity. These approaches help rebalance the power dynamics that often leave tenants and low-income residents vulnerable in housing markets dominated by large landlords and developers (Van De Bunt 2010).

One way to address these imbalances is through alternative housing models, such as cohousing, community land trusts, and cooperatives. These arrangements offer residents greater control over their living environments and can enhance affordability, resilience, and collective agency (Bates 2022). They also foster a sense of shared responsibility and long-term investment in community well-being.

Collective bargaining mechanisms, including tenant unions and grassroots organizations focused on housing rights, provide another avenue for empowering residents. These groups enable individuals who might otherwise be isolated to advocate for fair treatment, improved housing conditions, and policy reforms. By organizing collectively, tenants can negotiate more effectively and hold landlords and policymakers accountable (Bates 2022).

Urban planning can also foster community resilience. Designing inclusive, walkable neighbourhoods that encourage social interaction can help build informal support networks, which are especially important for those facing housing insecurity (Ramos-Vidal and de la Ossa 2024). Such environments promote not only physical health but also social cohesion and mental well-being.

Participatory policymaking is central to effective housing interventions. Inclusive consultation processes that engage residents, especially marginalized communities, ensure policies reflect lived experiences and local needs rather than being imposed from above. However, their success depends on enforceable diversity, equity, and inclusion (DEI) policies and mechanisms for community accountability (Goetz 2021, Teariki *et al.* 2025).

### Suitability

A healthy home is more than a roof over one’s head; it underpins physical, mental, and social well-being. It must provide safe energy for lighting and heating, maintain healthy indoor temperatures year-round, and ensure proper sanitation and ventilation to prevent disease and support respiratory health. It should also offer sufficient space for all household members and be accessible to people with disabilities, with necessary adaptations (Lakhani *et al.* 2025).

However, several developments have made it increasingly challenging to ensure housing suitability. One is demographic: as populations live longer, the demand for housing that accommodates older adults has grown significantly (GBD 2021 Diseases and Injuries Collaborators 2024). Another is environmental: elevated temperatures during heatwaves can lead to dangerous overheating in poorly ventilated dwellings, while increased precipitation may exacerbate moisture-related issues, such as mould growth (Vardoulakis *et al.* 2015).

Historically, local governments played a central role in regulating housing quality, operating within national frameworks. Yet, in many countries, this model has shifted towards a more hands-off, private-sector-led approach (Meijer and Visscher 1998). Developers, driven by cost-cutting incentives, often build smaller, poorly insulated dwellings using inexpensive materials. In some cases, deregulation has led to what researchers describe as ‘slow violence’, where substandard housing quietly undermines residents’ health over time (Chng *et al.* 2024). Large construction firms, acting as local monopolies or oligopolies, have lobbied to lower building standards, avoid obligations to provide affordable housing, and neglect the infrastructure necessary for healthy living. This section follows the same structure as the earlier ones on the other two pillars. As with housing security, we address the consequences of failure, in this case, the persistence of informal settlements, separately in a later section.

#### Legal and regulatory tools

Regulatory frameworks are essential for ensuring that housing meets basic safety, accessibility, and environmental performance standards. Governments can implement and enforce building regulations that address structural integrity, fire safety, thermal comfort, ventilation, and accessibility. These regulations should also promote climate resilience, requiring enhanced insulation, optimized ventilation systems, and universal design principles to accommodate people with disabilities (Lakhani *et al.* 2025).

The use of low-carbon and sustainable materials, with attention to embodied carbon in products such as concrete and steel, can reduce the environmental footprint of housing (Ruuska and Häkkinen 2015). However, regulations must be consistently enforced by local authorities to be effective.

Maintenance standards are crucial. Over 13% of European households report living in homes with leaking roofs, damp, or rotting structures (Eurostat 2025). Regulations can enforce proper maintenance, insurance, and repairs, especially in rental housing, where absentee landlords often neglect these responsibilities (Rose and Harris 2022). Landlord licensing and registration systems can uphold quality standards and increase accountability without relying solely on tenant advocacy (Jacobs 2015, More *et al.* 2017, Anderson *et al.* 2025).

None of these measures, however, will be effective unless those affected have access to justice, so they must be accompanied by legal aid for those unable to afford legal advice and representation (Moore and Dunning 2017). It is important to note that policies that put a high burden on tenants to take action to ensure their rights (e.g. gaining legal representation) can be ineffective or widen inequalities (Schwartz and Chu 2025), so any procedural or legal barriers to upholding tenants’ rights (e.g. in requesting repairs or challenging unlawful evictions) should be minimized.

#### Fiscal and financial measures

Governments can use financial tools to improve housing quality and sustainability. Direct financial support for retrofitting older buildings is especially important. Retrofitting measures, such as insulation upgrades; window glazing; high-efficiency heating, ventilation, and air conditioning (HVAC); and low-carbon heating technologies, can reduce energy consumption, lower utility costs, and improve resilience to extreme weather (Mata *et al.* 2015, Canada Green Building Council 2021).

Public investment can also support research and innovation in building techniques, including prefabricated construction and climate-resilient designs. Governments may fund demonstration projects in public housing to showcase high-quality, affordable, and sustainable construction (Kivimaa and Martiskainen 2018).

To support delivery, governments can invest in vocational training programmes and collaborate with industry associations to certify skills in modern construction techniques and sustainable practices (Brucker Juricic *et al.* 2021). Financial regulations can also mandate transparent contracts, warranty requirements, and accessible dispute resolution (Ashworth and Perera 2018). These measures create market pressures for construction firms to meet quality standards (Werna *et al.* 2022).

#### Planning and infrastructure measures

Planning systems play a foundational role in shaping housing suitability. Historically, local governments coordinated housing development within national regulatory frameworks. However, in many countries, this model has been replaced by private-sector-led approaches, often resulting in cost-cutting and reduced standards (Meijer and Visscher 1998).

Developers, particularly large firms with significant market or political power, may lobby to lower building standards, avoid obligations to provide affordable housing, and neglect the infrastructure needed for healthy living (Chng *et al.* 2024). This has led to poorly insulated, noisy housing that is ill-suited to extreme climatic conditions.

To counteract these trends, planning authorities must ensure that housing developments are integrated with community infrastructure, including transport, sanitation, and public spaces. Planning policies should also promote climate-resilient design, ensuring that new housing can withstand extreme weather and environmental stressors.

#### Community initiatives and participatory approaches

While technical standards and financial tools are essential, housing suitability also depends on how well homes reflect residents’ lived experiences. Community engagement in design and planning helps ensure that housing is not only structurally sound but also socially and culturally appropriate. Meeting the needs of diverse groups, including older adults and people with disabilities, requires inclusive consultation and design.

Participatory approaches are especially important where private-sector-led models dominate, and public oversight is limited (Meijer and Visscher 1998). Community involvement can also counteract ‘slow violence’ from deregulated planning, which has been linked to substandard housing and poor health outcomes (Chng *et al.* 2024).

The preceding sections have outlined how housing affordability, security, and suitability each contribute to health and well-being and how targeted policy interventions across legal, financial, planning, and community domains can strengthen these pillars. However, these domains do not operate in isolation. Their effectiveness is shaped, and often constrained, by broader systemic forces, including climate change, entrenched social inequalities, and the persistence of informal settlements and homelessness. These intersecting challenges demand a more integrated and adaptive policy response. In the following sections, we examine how climate-related risks compound housing vulnerabilities and how the most excluded populations, those living in informal settlements or experiencing homelessness, are disproportionately affected. These issues underscore the need for housing policies that address technical and economic barriers and promote resilience, equity, and inclusion at every level.

### Climate change and housing

Our changing climate, with rising temperatures, storms, flooding, and fires, has major implications for housing and health. The three housing pillars are interrelated, and climate hazards intensify problems of affordability, security, and suitability, shaped by social and economic inequalities. Policy must address both adaptation, to build resilience, and mitigation, to reduce housing’s carbon footprint. Climate impacts include building damage and higher maintenance costs, while residents face increased energy expenses to maintain thermal comfort (Gonçalves *et al.* 2024), disproportionately affecting low-income households in poorly insulated homes. Climate change also drives housing insecurity through displacement risks and rising insurance premiums in hazard-prone areas. Extreme weather can destroy housing stock, creating regional affordability crises that demand direct policy responses, including funding and measures to restore local supply (99% Invisible 2024).

Key climate mitigation measures related to housing and health include subsidies for energy efficiency and structural upgrades that improve the thermal comfort of homes, reducing energy costs for heating and cooling (Grant *et al.* 2021), as well as mitigating maintenance expenses and reducing insurance premiums. Additionally, tax reforms that incentivize investment in affordable housing and energy-efficient technologies, such as decentralized smart solar grids and virtual power plants, can alleviate the financial burden on households (Viggers *et al.* 2025).

Alongside upgrades, planning that takes into account urban climate science should seek to mitigate the impacts of increased building density in established areas with low tree canopy cover, such as the urban heat island effect, which is known to exacerbate urban health inequalities (Grislain-Letrémy *et al.* 2025). Green financial products, such as energy-efficiency loans and green mortgages, can provide the necessary capital for retrofitting projects (French and Kousky 2023, Hoeller *et al.* 2023, Gonçalves *et al.* 2024).

Addressing the risks posed by climate change through housing adaptation measures and integrating this with climate mitigation requires both physical and social infrastructure upgrades. Housing developments, particularly those serving vulnerable populations, should be retrofitted or constructed to meet climate-resilient standards, including improved insulation and passive design measures to protect against overheating, storm proofing, and flood mitigation systems (Brotas and Nicol 2016). Building codes should be updated and strictly enforced to reflect known and potential climate risks, and planning policies could require new developments, as well as bodies responsible for maintaining essential infrastructure, to consider critical environmental risks. A combination of the above measures can increase the resilience of households and communities.

When faced with a climate-related disaster, residents must either relocate, thereby increasing pressure on the limited housing stock elsewhere, or remain and risk devastation. Access to insurance coverage is crucial for protecting residents from the financial risks associated with climate change. However, even when households can afford insurance, insurers are increasingly unwilling to insure properties in floodplains, riverbanks, or coastal areas. Insurance products could include government-backed (re)insurance schemes to ensure continued protection for climate-vulnerable areas, as well as subsidies to lower premiums for vulnerable populations (French and Kousky 2023, Gonçalves *et al.* 2024). To avoid inadvertently encouraging construction in climate-vulnerable areas, appropriate planning and zoning policies are needed to restrict new development in high-risk areas.

In some cases, however, communities may need to be relocated, which will require financial support and coordinated action across multiple government departments and the private sector.

Importantly, whether addressing the conditions of informal settlements, the crisis of homelessness, or the direct threats of a changing climate, effective housing policies must be integrated with strategies that simultaneously tackle structural inequalities and build communities’ climate resilience.

### Informal settlements

While earlier sections have outlined specific policy responses to challenges in housing affordability, security, and quality, and their relationship to threats from climate change, these domains do not exist in isolation. Systemic inequities related to gender, disability, and socio-economic position fundamentally shape and exacerbate these domains of the housing system, including both internationally and within countries.

This section explores these critical intersections, examining how they manifest in relation to the acute vulnerability of people living in informal settlements and the complex crisis of homelessness.

An estimated 1.1 billion people live in informal settlements or slums worldwide, lacking basic amenities such as durable housing, safe water, and adequate sanitation (United Nations Economic and Social Council 2022). These settlements are overcrowded, with inadequate infrastructure and often high levels of violence, with adverse consequences for mental health (Cruz *et al.* 2021). Their inhabitants lack secure tenure and are at increased risk from extreme weather events such as floods, heatwaves, and storms (Borg *et al.* 2021) and from fires (Twigg *et al.* 2017). Poor construction, inadequate insulation, and inefficient cooling systems increase their susceptibility to climate-related health threats, which are discussed further below (Scott *et al.* 2017). This situation has arisen from inadequate investment in responses to rapid population growth in urban and periurban areas. Those living in these settlements have grown by 165 million in the past two decades (Reckford and Aki-Sawyerr 2023), despite a global commitment, in the Sustainable Development Goals, to reduce them (United Nations Economic and Social Council 2022).

There is still relatively limited research in low- and middle-income countries on how to improve the conditions in informal settlements (Turley *et al.* 2013, Weimann and Oni 2019), although lessons can be learnt from analogous situations, such as redevelopment in postindustrial communities (Tierney *et al.* 2024) or relocation of communities following natural disasters (Fussell and Lowe 2014). At its simplest, responses can be bottom-up or top-down. When implemented effectively, with meaningful community engagement, bottom-up solutions are more likely to be successful and sustainable (Lilford *et al.* 2017). However, whether this can be achieved depends on the distribution of power in each setting to avoid tokenistic consultations that leave those affected disenchanted and without any sense of ownership.

Successful solutions also involve much more than building new homes. Informal settlements often lie in climate-vulnerable areas, calling for investments in flood-resistant housing (Hussainzad and Gou 2024), while green infrastructure and renewable energy, such as solar panels, can increase sustainability. It will also be important to engage with infrastructure providers to provide electricity, clean water, and waste disposal, recognizing the challenges created by profit-maximizing private providers (Klein 2008), or the role of corruption, as revealed in a study of informal settlements that had managed to improve energy supply through connivance with electricity officials (Mensah 2022). There is, however, increasing evidence on how to implement solutions based on off-grid energy supplies (Runsten *et al.* 2018).

Informal settlements also suffer from fragmented governance, making it difficult to know who is responsible for what. Creating local resource centres delivering integrated services designed to improve health has been shown to improve some, but not all, outcomes in a cluster randomized controlled trial in India (More *et al.* 2017).

Legal protections are especially important, and governments should promote tenure security through land ownership registers, with studies in South America linking tenure provision for slum residents to improved health (Field 2005, Gandelman 2010). However, the effectiveness of such measures depends on the prevailing legal system, the authority of the government, the nature of evidence of tenure, and local custom and social attitudes (Reale and Handmer 2011).

As noted above, high-quality, climate-resilient housing tends to be comparatively expensive to build, so long-term success will depend on sustainable economic development and poverty reduction. The ways of achieving this go beyond the scope of this paper, but, at their simplest, require investment in people, emphasizing skills and health, as well as in places such as transport infrastructure, digital connectivity, and commercial buildings. These, in turn, require money, and, consistent with the bottom-up approach, this should include a substantial role for microcredit programmes that can offer small sums to start or expand small and medium enterprises (Gupta and Sharma 2023), which, by employing local service providers, brings wider benefits to the community, or to offer loans to enable households to invest in safer, healthier homes.

These measures can help to address the conditions of those living in informal settlements with some form of shelter, albeit inadequate, but at the extreme end of this spectrum of inequality lies acute homelessness.

### Homelessness

Finally, we turn to the most extreme failure of housing policy, homelessness. This can be defined not just by the absence of a physical roof, but by the lack of a safe, secure, and stable home (Amore *et al.* 2011). The Institute of Global Homelessness categorizes it into three categories: people without accommodation (e.g. sleeping on the streets and open or public places); people living in emergency accommodation, temporary shelters, hostels, or domestic violence refuges; and people with insecure/inadequate housing (e.g. living under threat of eviction or in unsafe/unacceptable conditions) (Institute of Global Homelessness 2019). The issues facing this last group are covered in earlier sections; here, we primarily refer to the first two categories under this framework.

Homelessness stems from a mix of social, economic, and political factors, including high housing costs, unemployment, mental health challenges, and lack of access to healthcare. A crisis such as job loss, illness, or domestic violence can quickly lead to housing insecurity. The consequences are severe. Those without a stable shelter are more exposed to violence, illness, and discrimination (Aldridge *et al.* 2018). Homelessness is also an important driver of vulnerability to extreme weather and the impacts of climate change (Bezgrebelna *et al.* 2021). Affected children often face disrupted education, increasing their risk of long-term poverty. Social stigma further isolates people, making recovery and reintegration difficult. All of these factors can contribute towards a harmful cycle of disadvantage.

The drivers of homelessness directly overlap and intersect with the root causes of the wider housing challenges discussed previously, including issues of housing affordability and insecurity, alongside stark and rising socio-economic inequalities in access to resources. However, responses are often piecemeal, addressing the symptoms of the underlying, interconnected causes rather than addressing them directly.

There are successes in tackling homelessness; notable examples include Finland (Dietz 2024) and New Zealand (Ombler *et al.* 2024). Rather than having to show that issues such as drug misuse have been addressed before being rehoused, homeless people are first given accommodation and then provided with support services. Solutions should be developed with those affected, as a recent Irish study highlighted, once again, that housing is much more than shelter but also a means of creating a new sense of identity and moving forward (Frazer *et al.* 2025).

## Discussion

As we noted earlier, this paper is intended as a resource for policymakers, and especially those concerned with health promotion, who are able to influence housing policy, as advocates, advisors, or policymakers. While it cannot replace in-depth expertise or context-specific analysis, it offers a framework for understanding how housing affects health. It also reflects the expanding scope of health promotion to include social, environmental, commercial, and political factors, highlighting housing’s role in both contributing to and adapting to climate change.

Our paper complements existing frameworks for understanding the association between housing and health. Like the WHO Housing and Health Guidelines, it underscores housing as a determinant of health and promotes evidence-based standards for ventilation, thermal comfort, and safety (World Health Organization 2018). However, it extends beyond technical quality by elevating affordability and security as equal pillars, which the WHO addresses only indirectly. Similarly, it shares common ground with Housing First approaches in prioritizing stability and security as prerequisites for health, yet differs in scope: Housing First focuses primarily on homelessness, whereas our framework situates homelessness within a broader systemic structure encompassing affordability, suitability, and climate resilience (Dietz 2024). Our framework also reflects the equity and sustainability principles embedded in the Sustainable Development Goals, but operationalizes them through specific policy levers, specifically legal, financial, planning, and community-based measures, rather than aspirational targets. Its most distinctive contribution lies in adopting a systems-thinking perspective and offering a flexible menu of policy options tailored to diverse governance contexts, in contrast to the prescriptive nature of many existing guidelines.

Our key message is that the domains of affordability, security, and suitability, taken together, highlight the complexity of housing as a determinant of health. Such a holistic approach is especially important in the light of intersecting challenges of climate change, informal settlements, and homelessness. Moreover, while a full consideration would require more space than is available to us here, a health promotion perspective will also consider the socio-economic factors that shape affordability, tenure security, and suitability, noting how those already disadvantaged are often most vulnerable to the effects of climate change.

We noted, at the outset, that we would avoid detailed policy recommendations, but we can offer some broad principles. First, policymakers should have a comprehensive understanding of the problems they face. Our framework seeks to help them, encouraging an exploration of our three pillars, the foundations on which they sit, and the policy levers available. Box 1 provides a checklist for health promotion practitioners to adopt a broad perspective. Only by addressing these issues in an integrated and context-sensitive manner can housing policy truly support health and equity.

Box 1. Checklist for health promotion and protection in the housing system **Affordability**

- Is housing in this community affordable relative to income levels?
- What market failures (e.g. lack of transparency and developer dominance) are affecting affordability?
- Are there effective public interventions (e.g. subsidies and social housing) in place?
- How are changes such as gentrification impacting vulnerable populations?

**Security of Tenure**

- Do tenants have adequate legal protections against eviction?
- Are emergency supports (e.g. rent assistance and eviction moratoria) available during crises?
- Are there mechanisms for collective action by tenants or advocacy?
- Is access to legal aid sufficient to protect housing rights?

**Suitability and Quality**

- Are homes safe, well ventilated, and thermally comfortable?
- Do building regulations ensure health-promoting standards?
- Is there support for retrofitting older homes to improve health outcomes?
- Are homes accessible for people with disabilities and older adults?

**Climate Resilience**

- Are homes protected against climate hazards such as flooding and heatwaves?
- Are energy-efficient upgrades and green infrastructure being promoted?
- Is insurance accessible and affordable for climate-vulnerable households?

**Informal Settlements and Homelessness**

- What health risks are present in informal housing or slums?
- Are there community-led upgrading initiatives or infrastructure investments?
- Are there policies for addressing homelessness?

**Equity and Inclusion**

- Are housing policies addressing the needs of youth, disabled, Indigenous, and other marginalized groups?
- Is there meaningful community participation in housing decisions?
- Are health literacy and public awareness around housing being promoted?

**Governance and Integration**

- Who holds responsibility for housing policy at local and national levels?
- Are housing, health, and planning sectors working together effectively?
- Are policies evidence-informed and adapted to local contexts?

Second, they must take into account context, which, again, our framework should help address. This is evident in the foundations. Legal protections safeguard tenants’ rights and housing quality, especially in insecure or informal settings. Community participation enhances legitimacy and ensures policies reflect lived experience. Evidence-informed policy enables adaptive responses to challenges such as climate change. Access to justice is essential for the enforcement of rights, particularly for marginalized groups. Together, these elements create the conditions for housing systems that support health and resilience, at all times recognizing the importance of addressing social determinants of housing and health.

Third, they must identify who can turn their ideas into practice. This requires a systems analysis, identifying who can do what, in what circumstances, what constraints they face, and how they interact with others (Checkland 2000). Effective governance structures are key (Greer *et al.* 2016). In particular, they must identify the appropriate level of government at which to act, as responsibilities vary between federal and centralized systems.

We must recognize that our approach has some limitations, reflecting the explicit choices that we made. First, we adopted a broad approach to a complex problem, sacrificing depth for breadth. Second, we adopted an iterative exploratory approach rather than an *a priori* systematic one, following leads as they arose. We accept that this may introduce bias, but the alternative, such as one or more umbrella reviews, risks perpetuating disciplinary siloes and excluding important considerations. Third, we did not conduct quantitative modelling, so we cannot estimate health impacts or cost-effectiveness. Fourth, although we address vulnerable groups, their needs require deeper, context-specific analysis. Finally, given the pace of change in climate and housing, policy options must be regularly revisited to stay relevant.

## Conclusion

There is an urgent need for integrated policy responses to the complex housing, health, and equity challenges facing populations globally. A range of effective measures already exists, including regulatory, fiscal, financial, urban planning, and system-level reforms. This paper’s framework aims to support policymakers in exploring these options to improve housing affordability, security, and quality, thereby advancing public health. Achieving healthy housing for all requires coordinated action across governments and the private sector at local, national, and global levels. The potential to strengthen community resilience, reduce health inequalities, and address climate-related risks makes housing a critical priority for public health.

## Data Availability

This perspective is based on published literature, all of which is referenced in the text
